# Template synthesis of the Cu_2_O nanoparticle-doped hollow carbon nanofibres and their application as non-enzymatic glucose biosensors

**DOI:** 10.1098/rsos.181474

**Published:** 2018-12-12

**Authors:** Yingjie Li, Renhao Cai, Renjiang Lü, Lidi Gao, Shili Qin

**Affiliations:** College of Chemistry and Chemical Engineering, Qiqihar University, Qiqihar, Heilongjiang 161006, People's Republic of China

**Keywords:** template synthesis, Cu_2_O nanoparticles, doped, hollow carbon nanofibres, biosensors

## Abstract

The cuprous oxide nanoparticle (Cu_2_O NP)-doped hollow carbon nanofibres (Cu_2_O/HCFs) were directly synthesized by the anodic aluminium oxide (AAO) template. The doped Cu_2_O NPs were formed by *in situ* deposition by direct reduction reaction of precursor carbonization in thermal decomposition and could act as functionalized nanoparticles. The synthesized Cu_2_O/HCFs were characterized in detail by transmission electron microscopy (TEM), scanning electron microscopy (SEM), X-ray diffraction (XRD), X-ray photoelectron spectroscopy (XPS), Raman spectroscopy and inductively coupled plasma mass spectrometry (ICP-MS). The results reveal that Cu_2_O/HCFs have a tubular structure with an average diameter of approximately 60 nm. The shape of the Cu_2_O/HCFs is straight and Cu_2_O NPs are uniformly distributed and highly dispersed in HCFs. Cu_2_O/HCFs have good dispersibility. The electrochemical activity of Cu_2_O/HCFs was investigated by cyclic voltammetry (CV), the glucose sensors display high electrochemical activity towards the oxidation of glucose. Cu_2_O/HCFs can effectively accelerate the transmission of electrons on the electrode surface. Cu_2_O/HCFs are applied in the detection of glucose with a detection limit of 0.48 µM, a linear detection range from 7.99 to 33.33 µM and with a high sensitivity of 1218.3 µA cm^−2^ mM^−1^. Moreover, the experimental results demonstrate that Cu_2_O/HCFs have good stability, reproducibility and selectivity. Our results suggest that Cu_2_O/HCFs could be a promising candidate for the construction of non-enzymatic sensor.

## 1. Introduction

Owing to the special structure, good stability, unique electronic properties and the extraordinary mechanical properties, carbon-based materials have attracted considerable attention since they were discovered, and are widely used in many important technological fields such as catalysis [1], sensors [2], adsorbents [3] and electronic devices [4]. Among numerous carbon-based materials, one-dimensional hollow carbon-based materials have attracted more and more scientific and technological interest owing to their excellent properties such as high aspect ratio, light weight, high thermal conductivity, excellent electroconductibility and other superior characteristics [5].

To improve the practical application of one-dimensional materials, in recent years, researchers have combined metal and metal oxides with one-dimensional carbon-based materials [6,7]. The incorporation of metal or metal oxide nanoparticles with one-dimensional carbon-based materials may produce the synergistic effects and may lead to the improved property of the composites [8,9]. At present, such studies have been reported. For example, Zn ferrite/multi-walled carbon nanotube (Zn ferrite/MWCNT) composite was prepared [10]. The Zn ferrite/MWCNT composite showed the addition of low amount, low coating thickness and enhanced EM-wave absorption performance. Unique morphology Fe_3_O_4_/Fe-carbon nanotube (CNT) nanocomposites at room temperature by a facile chemical synthesis method [11]. The capacitance of Fe_3_O_4_ could be extremely improved by dual conduction system containing CNTs and Fe. Ni-TiO_2_/carbon nanotube photocatalysts were synthesized by a simple method [12]. The CNTs could be applied as useful photocatalytic support for the fixation of TiO_2_. Hydrogen evolution was enhanced by the Ni loading on the TiO_2_ nanocrystallites supported on the carbon nanotube. Combined metal or metal oxides with one-dimensional carbon-based materials have attracted more and more scientific interest.

Among various metal oxides, Cu_2_O, as an important class of p-type semiconductor, has been paid much attention in enzyme-free glucose sensors in recent years owing to its high electrocatalytic activity, low cost and good stability, and it is a promising candidate in the fabrication of electrochemical materials, [13] and advantages of Cu_2_O have been intensively investigated [14]. For example, the Cu/Cu_2_O nanocluster-deposited carbon spheres have been synthesized through a layer-by-layer assembly method and subsequent *in situ* self-reduction process. The results showed that the Cu/Cu_2_O nanoclusters are homogeneously anchored onto the carbon spheres. The double-shelled Cu/Cu_2_O/CSs showed remarkable electrocatalytic activity toward glucose oxidation including two linear ranges with high selectivity of 63.8 and 22.6 µA cm^−2^ mM^−1^ as well as good stability and repeatability [15]. Yazid *et al*. reported a highly sensitive and selective glucose sensor based on cuprous oxide/graphene nanocomposite-modified glassy carbon electrode (Cu_2_O/graphene/GCE). The proposed sensor was successfully applied for the determination of glucose concentration in real human blood samples [16]. A type of nanospindle-like Cu_2_O/straight multi-walled carbon nanotube (SMWNT) nanohybrid-modified electrode for sensitive enzyme-free glucose detection has been fabricated, the as-prepared nanospindle-like Cu_2_O/SMWNT nanohybrids exhibit much higher electrocatalytic activity on the oxidation of glucose than the SMWNTs or Cu_2_O alone as the electrode-modifying material. More importantly, the nanohybrid-modified electrodes also show good stability, reproducibility and high resistance against poisoning by chloride ion and the commonly interfering species such as ascorbic acid, dopamine, uric acid and acetamidophenol. These good analytical performances make the nanospindle-like Cu_2_O/SMWNT nanohybrids promising for the future development of enzyme-free glucose sensors [17].

Recently, research on Cu_2_O-doped carbon-based materials has gradually increased. Various morphologies of Cu_2_O-doped carbon-based materials have been prepared by several different methods, such as chemical vapour deposition method, hydrothermal method, solvothermal method and wet chemical method [18–22]. These materials show excellent performance in solar cells, sensors and catalysis and other fields; however, there are some defects, such as uneven nanoparticle size, poor dispersion, carbon nanotube agglomeration and other issues. The unique structure of anodic aluminium oxide (AAO) template makes them very promising hosts for preparing one-dimensional nanomaterials. The precursor has been applied to backfill the template, which has resulted in the formation of one-dimensional nanostructures. Considering the composition of AAO template, it is believed that metal doped or decorated in the prepared one-dimensional nanostructures would be obtained through the *in situ* deposition reaction of AAO template. Therefore, we require a careful synthetic strategy to fabricate Cu_2_O-doped HCFs with a high surface-to-volume ratio and expect to improve the electrochemical activity of HCF film enzyme-free glucose sensors, which is still a challenging work.

Herein, we have proposed a simple and effective technique for preparing novel hollow carbon nanofibres (HCFs) with highly dispersed Cu_2_O nanoparticles by using the one-step direct AAO template route, which employed glucose as the carbon source and copper acetate (CuAc_2_) as the dopants. To investigate the structure and morphology of Cu_2_O/HCFs, scanning electron microscopy (SEM), transmission electron microscopy (TEM), X-ray diffraction (XRD), Raman spectra and X-ray photoelectron spectroscopy (XPS) were carried out. Cyclic voltammetry (CV) was used to evaluate the electrochemical detection of the Cu_2_O/HCFs-modified electrode towards glucose.

## 2. Material and methods

### 2.1. Materials

Glucose, phosphoric acid, Na_2_CO_3_ and HNO_3_ (AR, Kermel Ltd, China); copper acetate and perchloric acid (AR, Aladin Ltd, China); sodium hydroxide (AR, Jinli Ltd, China), high purity aluminium (99.999%, Mengtaiyouyan Technology Development Center, China); anhydrous ethanol (AR, Fuyu Fine Chemical Ltd, China) were used.

### 2.2. The preparation of AAO template

AAO templates were prepared by two-step anodization. High purity (99.999%) aluminium plates were ultrasonically cleaned in the mixed solution of acetone and alcohol (v/v = 1 : 1) for 30 min. All aluminium plates were annealed at 500°C for 2 h in a conventional furnace and degreased with acetone. The high purity aluminium plates were anodized in a 0.3 M oxalic acid solution under an applied voltage of 40 V and at a temperature around 4°C for 6 h. After accomplishing the first anodizing stage, alumina layers were removed by wet etching in aqueous solution of 6 wt% phosphoric acid and 1.8 wt% chromic acid at 70°C for 20 min. The textured Al plates were anodized again for 72 h under the same conditions as for the first anodizing. A subsequent etching treatment was carried out in a 6 wt% phosphoric acid solution at 40–50°C for 1 h, followed by washing and drying.

### 2.3. The preparation of Cu_2_O/HCFs

Glucose (10.00 g) and different mass CuAc_2_ (0.50, 1.00, 2.00 and 4.00 g) were dissolved in 100 ml deionized water to obtain the corresponding precursors, the fabricated precursors were labelled as H-1, H-2, H-3 and H-4, respectively. After magnetic stirring for 30 min, the AAO membranes on Al substrate were placed in a home-made device, with the precursor in a separating funnel, and the reactor was subsequently evacuated. After opening the funnel cock, the nano-channels of the AAO templates were filled with the precursor depending on the atmospheric pressure. The templates were then removed from the vacuum container. After drying at room temperature for 6 h in an ambient environment and removal of surface residues [23], the templates were calcined in the N_2_ atmosphere for 3 h at different temperatures (400, 500 and 600°C). After slow cooling to room temperature, the Cu_2_O/HCFs were obtained after removing the alumina template by etching with the 6 M NaOH solution.

### 2.4. The preparation of Cu_2_O/HCFs/GCE

The modified electrode was prepared as follows. Five microlitres of the suspension with dispersed Cu_2_O/HCFs was coated on the pre-treated glassy carbon electrode and dried at room temperature. Before modification, the bare GCE was polished with 1, 0.5 and 0.03 µm alumina slurry and then washed ultrasonically in deionized water, 50% (v/v) HNO_3_ solution, ethanol and water for 6 min [24].

### 2.5. Electrochemical measurements

Electrochemical measurements were carried out on a CHI660E Electrochemical Workstation with a conventional three-electrode system composed of a platinum wire as an auxiliary electrode, a saturated Hg/Hg_2_Cl_2_ (SCE) as a reference electrode [15] and the modified electrode as a working electrode.

## 3. Results

**Figure 1.**
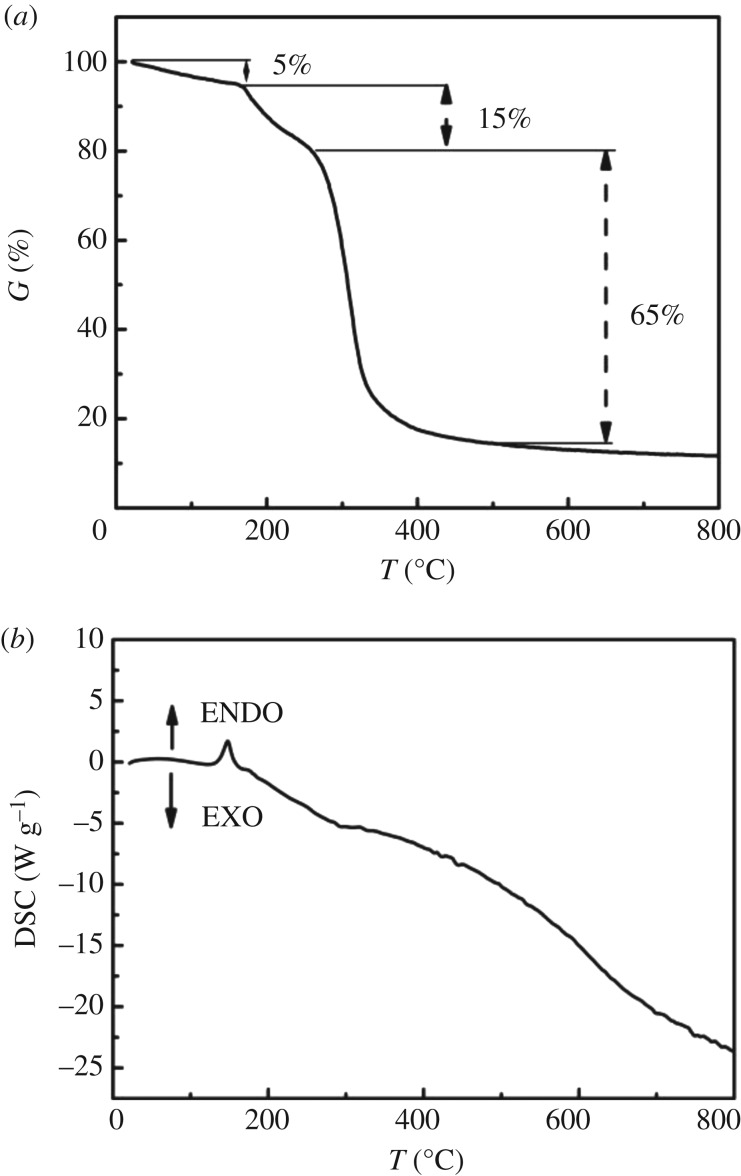
TG (*a*) and DSC (*b*) thermoanalytical response curves of precursor.

Thermogravimetric (TG) curves of the precursor are displayed in figure 1. They revealed that the obvious weight loss occurred from room temperature to 170, 170 to 260 and 260 to 550°C. The first step of 5% weight loss corresponds to the evaporation of water from the precursor, and the second step of 13% weight loss contributes to complete decomposition of copper acetate at the higher temperature with their corresponding endothermic peaks, which is in agreement with the composition of the precursor, as shown in the following reaction equation:

$${\mathrm{Cu}{(\mathrm{CH}}_{3}\mathrm{OO})}_{2}\to {\mathrm{CH}}_{4}+{\mathrm{CO}}_{2}+\mathrm{CuO}.$$

The third step of 65% weight loss for precursor may be due to the decomposition of glucose in the precursor. Above 550°C, the weight loss of the precursor remains unchanged. Glucose reduces CuO to Cu_2_O. According to the TG data, 600°C was used as the calcination temperature of the products.

**Figure 2.**
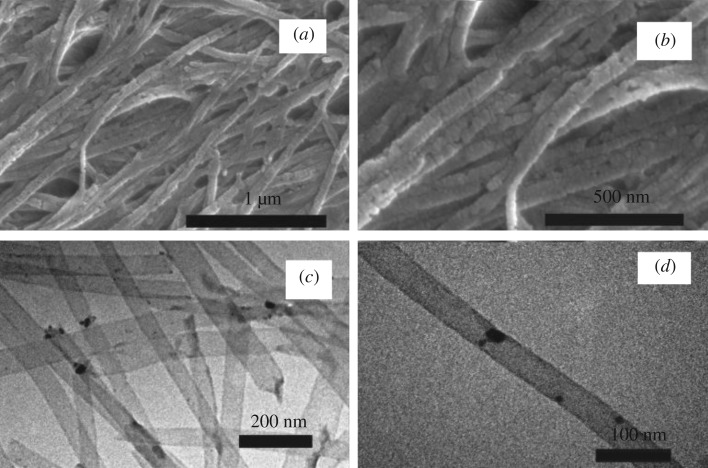
SEM images of the Cu_2_O/HCFs: (*a*) large-area view and (*b*) small-area view, TEM images of the Cu_2_O/HCFs: (*c*) lots of Cu_2_O/HCFs, (*d*) a single Cu_2_O/HCFs.

The surface morphologies of the as-synthesized Cu_2_O/HCFs as imaged by SEM are shown in figure 2*a*,*b*. It can be observed from the photos that the carbon nanomaterials are overlapped with each other to form a large-area reticular morphology, the carbon nanomaterial is uniform in size, and the shape is straight. The prepared samples have a fibre structure with the uniform outer diameter of approximately 60 nm. The diameter and length of the samples mainly depend upon the porous nature of the AAO membrane (SEM images of the top view, electronic supplementary material, figure S1). Figure 2*c*,*d* shows the TEM images of Cu_2_O/HCFs. One can see that almost all the Cu_2_O/HCFs are hollow structures and have good monodispersity. Cu_2_O NPs in the HCFs are highly dispersed and have no aggregation and the average size of Cu_2_O NPs is about 21.5 nm. SEM and TEM display Cu_2_O/HCFs outside the diameter, from which the average diameter of Cu_2_O/HCFs can be 60 nm. This diameter is consistent with the pore diameter of the AAO templates.

**Figure 3.**
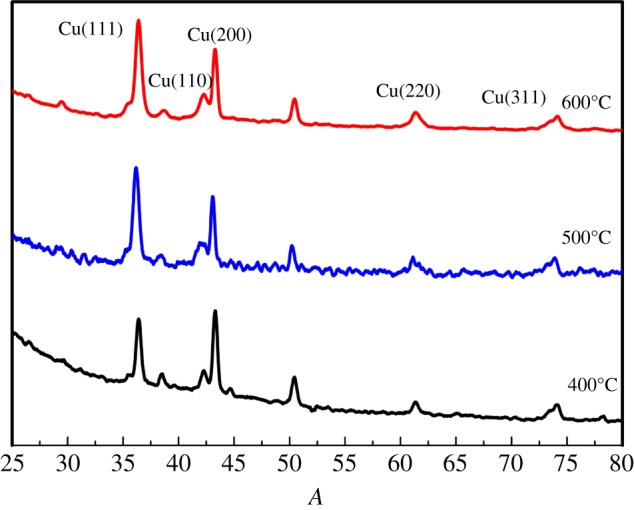
XRD patterns of Cu_2_O/HCFs. Those were prepared at 400, 500 and 600°C for Cu_2_O/HCFs.

**Figure 4.**
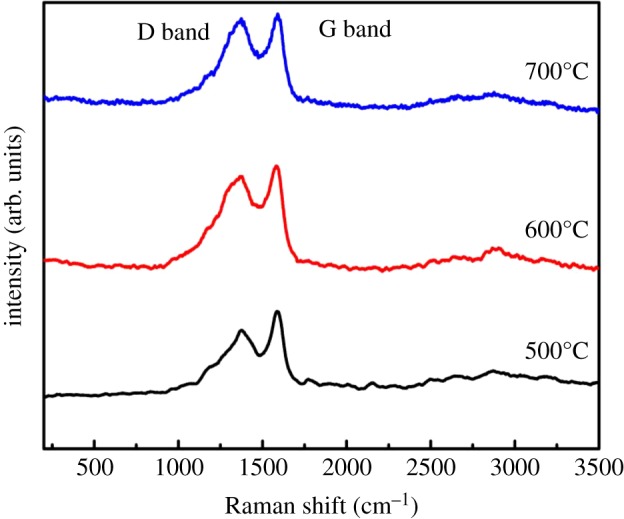
Raman spectra of Cu_2_O/HCFs. Those were prepared at 400, 500 and 600°C for Cu_2_O/HCFs.

The crystal structure of the as-synthesized catalysts was analysed by the XRD technique and their patterns of calcination at different temperatures are given in figure 3. All relatively intense diffraction peaks with 2θ angles of 36.2°, 43.5°, 62.4° and 74.7° correspond to the crystal planes of (111), (200), (220) and (311) of Cu matching well with the standard XRD pattern for Cu_2_O of face-centred cubic lattice [25]. No peaks of impurity (such as Cu) were found in the XRD patterns, indicating that the obtained particles are pure cubic phase Cu_2_O with high crystallinity. It must also be mentioned that the peaks broaden obviously; this indicates that smaller Cu_2_O nanoparticles were formed in HCFs. The characteristic diffraction peak of graphitized carbon was found at 2θ angles of 26.02°and 42.3°; according to the literature [26], the (002) (100) crystal face was assigned to carbon. Owing to the decomposition of glucose in the precursor to form hollow carbon nanostructures after high temperature calcination, Cu_2_O nanoparticles appear in HCFs. The crystallite size was around 21.5 nm using the Scherrer formula; this is consistent with the observation from TEM. Figure 4 shows the Raman spectra of the samples calcined at 400, 500 and 600°C. The spectrum of our sample contains G band at 1584 cm^−1^ and D band at 1352 cm^−1^. The D band represents the disordered graphite associated with defects and amorphous carbon, whereas the G band represents the ordered graphite corresponding to the stretching mode of the C–C bonds in the graphite plane. The D band is assigned to the breathing mode of A_1 g_ symmetry due to the phonon interaction near the K zone boundary, while the G band is attributed to the E_2 g_ phonon mode of the sp^2^ bonded carbon atoms [27–29]. With the calcination temperature rising, Raman spectra undergo significant changes. Specifically, the G band broadened significantly and displayed a shift to higher frequencies (blue shift), G band moved from 1112 cm^−1^ to 1154 cm^−1^ and the D band D peak intensity increased from 151.03 to 178.71. At 600°C, the peak Intensity ratio (*I*_G_/*I*_D_) was 1.22, crystalline carbon content gradually increases and indicates that the amorphous carbon transforms into graphitized carbon, which is consistent with the XRD results.

**Figure 5.**
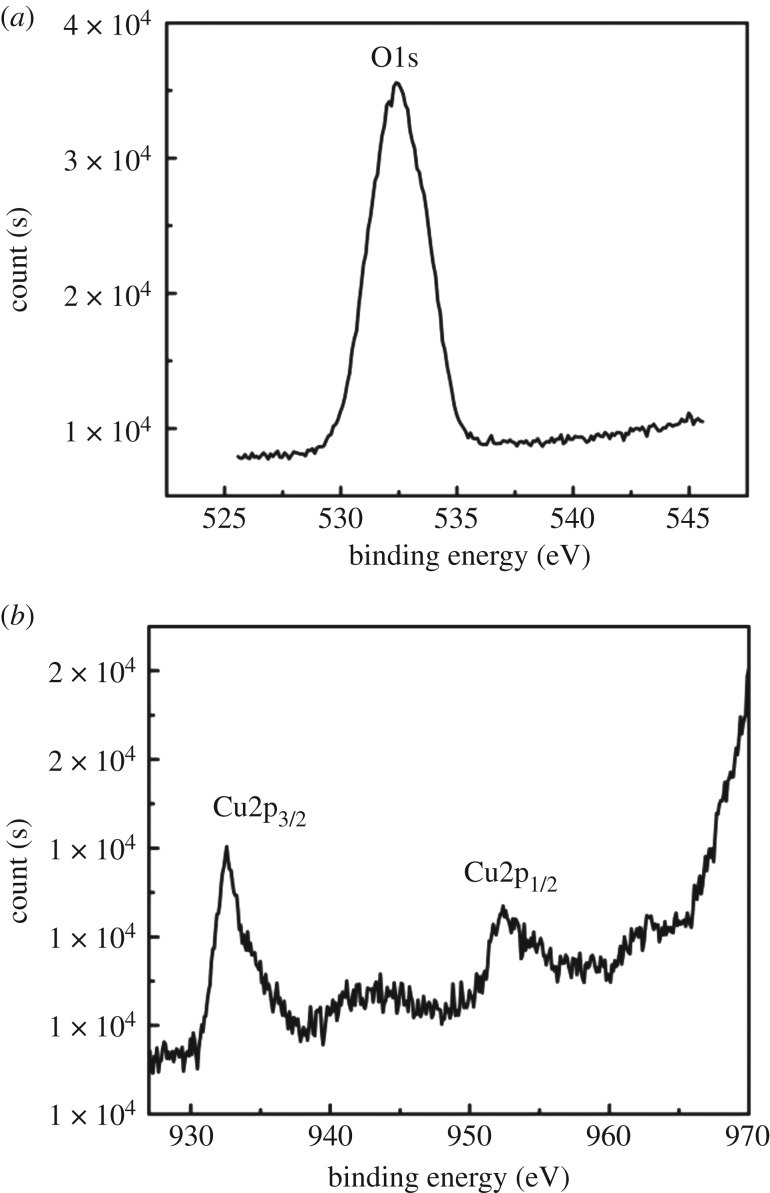
XPS spectra of Cu_2_O/HCFs. (*a*) O1s, (*b*) Cu2p_3/2_ and Cu2p_1/2_.

Figure 5*a*,*b* is O1s and Cu2p X-ray energy spectrum, respectively. As can be seen from figure 5*a*, the peak at 532.38 eV was assigned to O1s of Cu_2_O. As we know, the binding energy of O1s in the crystal lattice is 528.5–529.7 eV, and the binding energy of absorbed oxygen is 530.54–533.77 eV [30]. Owing to the combination of copper and oxygen in the precursor, the O1s peak appears in figure 5*a*. As shown in figure 5*b*, the peaks at 932.58 and 953.38 eV were, respectively, assigned to Cu2p_3/2_ and Cu2p_1/2_ of Cu_2_O, suggesting the presence of Cu_2_O [31]. It is reported in the literature that a satellite peak in the XPS spectrum of Cu2p indicates the presence of Cu(II) in the sample, unobvious satellite peaks appear in the figure 5*b*, of Cu(II) in the sample, unobvious satellite peaks appear in figure 5*b*, this means the presence of low content of Cu(II) in the sample [32]. The results of XPS are in consistent with the observation from XRD, it is further explained that the metal nanoparticles in the material are mainly Cu_2_O particles.

**Figure 6.**
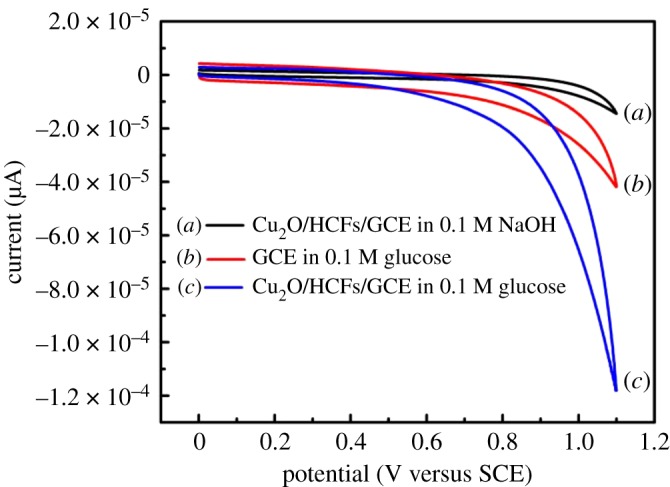
The CV curves of different condition at 100 mV s^−1^ (*a*) Cu_2_O/HCFs/GCE in the NaOH base solution, (*b*) the bare GCE in the NaOH solution containing 0.1 M glucose, (*c*) Cu_2_O/HCFs/GCE in the NaOH solution containing 0.1 M glucose.

To investigate the electrochemical properties of Cu_2_O/HCFs, the CV studies were performed in glucose solution at a scan rate of 100 mV s^−1^ and potential range from 0 to +1.1 V concerning the electrode. Zhang *et al*. [33] investigated the effect of pH value on the glucose electrochemical oxidation by Cu_2_O/HCF-modified electrode. The result showed that it is helpful to the mutarotation of glucose at high pH value and increasing current. Because glucose is easily oxidized under alkaline conditions, we choose 0.1 M NaOH for non-enzymatic glucose detection. Figure 6*a*–*c* severally displays the cyclic voltammograms (CVs) of the bare GCE, Cu_2_O/HCFs/GCE in 0.1 M NaOH with and without the presence of glucose at room temperature; *a* is the CV curve of Cu_2_O/HCFs/GCE in the blank NaOH base solution, *b* is the CV curve of the bare GCE in the NaOH solution containing 0.1 M glucose and *c* is the CV curve of the Cu_2_O/HCFs/GCE in the NaOH solution containing 0.1 M glucose. From curves *b* and *c*, it can be seen that an oxidation process started at *ca* +0.5 V and reached a peak at about +1.1 V on the modified electrodes, but the modified electrode has the more obvious electrochemical response to glucose. Comparing *a* and *b* curves, while no peak has been observed in the CV curve in the absence of glucose, a dramatic change was observed at the electrode when glucose was added. Anodic peak current of Cu_2_O/HCFs/GCE apparently enlarged at 0.8 V. This illustrated that the Cu_2_O/HCFs/GCE have the electrochemical ability of glucose oxidation, which corresponded to the irreversible glucose oxidation due to the conversion of Cu(II) to Cu(III). The possible reaction could be explained by the following equations:

$$\mathrm{CuO}+3H_{2}O\to 2\mathrm{Cu}{\left(\mathrm{OH}\right)}_{2}+H_{2},$$

$${\mathrm{Cu}(\mathrm{OH})}_{2}\to \mathrm{CuO}+H_{2}O,$$

$$\mathrm{CuO}+OH^{-}\to \mathrm{CuOOH}+e^{-}\to {\mathrm{Cu}(\mathrm{OH})}_{4}^{-}+e^{-}$$

$$\;\mathrm{and}\;\mathrm{Cu}(\mathrm{III})+\mathrm{glucose}+e^{-}\to \mathrm{gluconolactone}+\mathrm{Cu}(\mathrm{II}).$$

**Figure 7.**
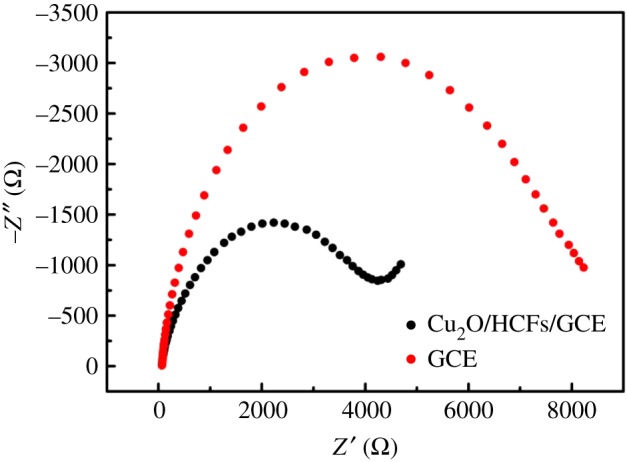
Nyquist plots for Faradic impedance measurement of bare GCE (red curve) and Cu_2_O/HCFs/GCE (black curve) in 0.1 mol l^−1^ NaOH containing 10 mmol l^−1^ K_3_[Fe(CN)_6_]/K_4_[Fe(CN)_6_]. The frequency range is from 0.01 Hz to 100 kHz and the amplitude is 0.21 mV.

Cu(III) is generated on the Cu_2_O surface rapidly and the oxidized glucose is converted to gluconic acid. Also, the conversion of Cu(III) into Cu(II) species gives rise to the increase in the oxidation peak current and the decrease in the reduction peak current [34]. The formation of Cu(III) species not only leads to high catalytic activity but also plays the role of an electron transfer mediator. Moreover, the cubic Cu_2_O has the characteristics of the large specific surface area and is conducive to electrochemical reactions [35]. Figure 7 shows Nyquist plots for Faradic impedance measurement of bare GCE (red curve) and Cu_2_O/HCFs/GCE (black curve) in 0.1 mol l^−1^ NaOH containing 10 mmol l^−1^ K_3_[Fe(CN)_6_]/K_4_[Fe(CN)6]. The frequency range is from 0.01 Hz to 100 kHz and the amplitude is 0.21 mV. When the electrode was modified with Cu_2_O/HCFs, the diameter of the semicircle became significantly smaller, indicating that Cu_2_O/HCFs can effectively accelerate the transmission of electrons on the electrode surface. The results of the electrochemical impedance spectroscopy (EIS) of the glassy carbon electrode and the modified electrode are consistent with the results of CV.

**Figure 8.**
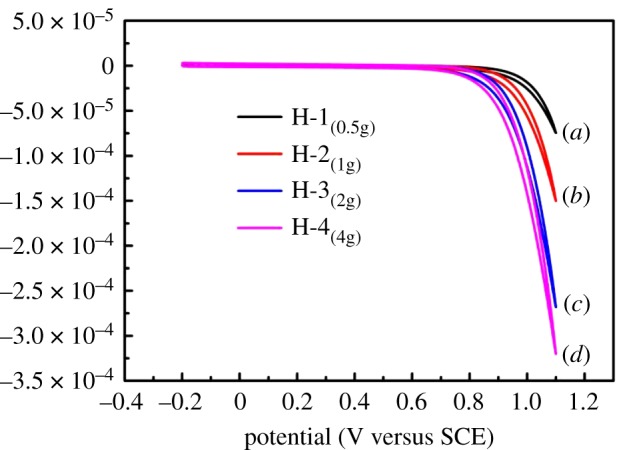
The CV curves of different products at 100 mV s^−1^ in the 0.1 M NaOH solution containing 0.1 M glucose. (*a*–*d*) is the curve of H-1, H-2, H-3 and H-4 samples, respectively.

The Cu contents of the H-1, H-2, H-3 and H-4 samples were obtained by ICP-MS, and the amounts of Cu in the products are listed in table 1. The relationship between the peak current value and the contents of copper was analysed. It can be seen from table 1 that as the content of copper acetate in the precursor increases, the content of Cu in the sample is also increasing, but the content of Cu does not increase linearly. When the mass of copper acetate in the precursor is increased from 0.5 g to 2 g, the content of Cu in HCFs changes obviously. When the mass of copper acetate in the precursor is increased from 2 g to 4 g, the content of Cu in MHCFs is no longer changed obviously. This coincides with the change in the peak current of figure 8. The reason is that the content of glucose is reducing; however, the content of glucose in the precursor is constant, and its reducing ability is also limited, only a certain amount of copper acetate can be reduced, therefore, according to the data from table 1, the optimum doping amount of copper acetate is 2 g.

**Table 1.** The amount of Cu in the products.

sample	content of Cu in the products (wt%)
H-1	7.43
H-2	13.00
H-3	20.65
H-4	22.52

**Figure 9.**
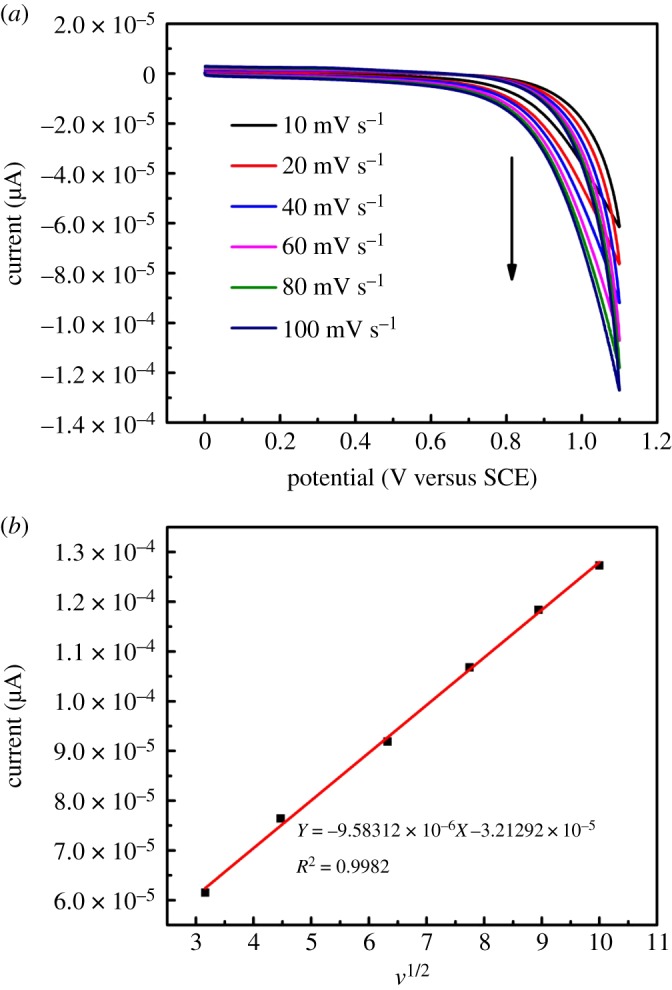
(*a*) CVs of the Cu_2_O/HCFs/GCE in the 0.1 M NaOH solution with 5.0 mM of glucose at different scan rates (10, 20, 40, 60, 80, 100 mV s^−1^, respectively). (*b*) The linear dependence of oxidation peak current with the scan rate.

**Figure 10.**
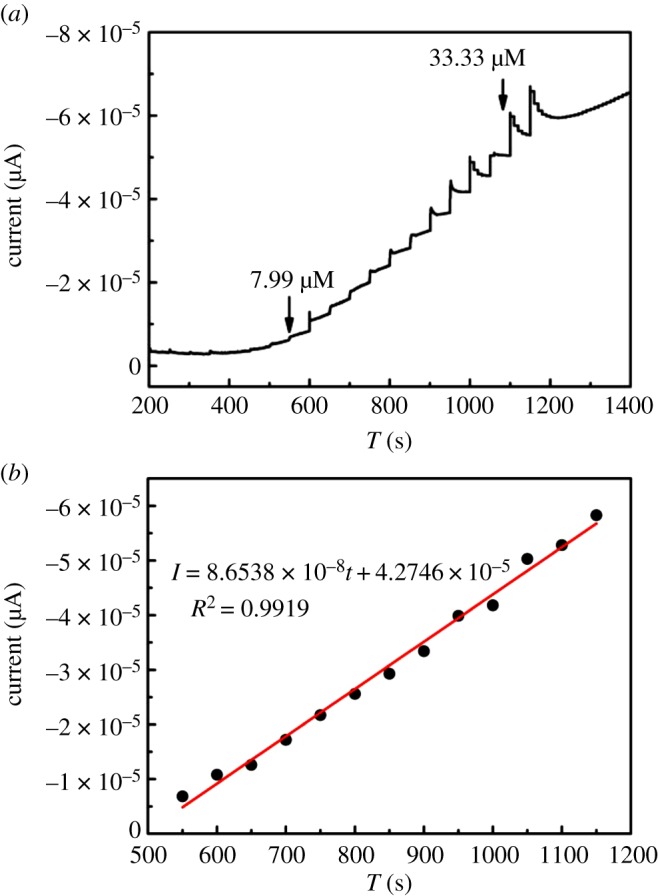
(*a*) Amperometric responses of the Cu_2_O/HCF-modified electrode after successive injection of 40 mM glucose in the 0.1 M NaOH solution at the applied potential of 1.1 V. (*b*) Plot of the catalytic current versus glucose concentration.

To further discuss the electrochemical properties, the CVs of Cu_2_O/HCFs electrode at different scan rates ranging from 10 to 100 mV s^−1^ in the potential window of 0–1.1 mV in the 0.1 M NaOH solution containing 1.0 mM glucose. The oxidative peak current increased with the increasing scan rate in the range of 10–100 mV s^−1^, as shown in figure 9*a*. A linear relationship with a linear regression equation

$$I(A)=-9.58\times 10^{-6}v-3.21\times 10^{-5}\;(R^{2}=0.9982),$$

between the oxidation peak current of glucose and the square root of scan rate is observed, as shown in figure 9*b*. From the results we have obtained, one can conclude that the electrochemical kinetic is controlled by the adsorption of glucose. Figure 10*a* illustrates the amperometric responses of the Cu_2_O/HCFs/GCE upon successive addition of various concentrations (7.99–33.33 µM) of glucose at a work potential of 1.1 V. To achieve a homogeneous glucose concentration instantly, the solution was vigorously stirred to ensure good distribution of electrolyte and glucose. Upon the addition of glucose, the Cu_2_O/HCFs/GCE reached the dynamic equilibrium within 6 s, indicating a very fast amperometric response of the modified electrodes. Figure 10*b* shows the calibration curve of different concentrations of glucose on the modified electrodes. As can be seen from figure 10*b*, the Cu_2_O/HCFs-modified electrode displays a good linear range from 7.99 to 33.33 µM and the linear regression equation can be expressed as

$$I=8.6538\times 10^{-8}C-4.2746\times 10^{-5}\;(R^{2}=0.9919),$$

where *I* and *C* represent the response current and glucose concentration, respectively. With a high sensitivity of 1218.3 µA cm^−2^ mM^−1^, the low limit of detection (LOD) was 0.48 µM by *S*/*N* = 3, where *S* and *N* are the standard deviation of the background current and slope of the calibration curve, respectively. Obviously, the Cu_2_O/HCFs-modified electrode here possesses favourable analytical properties. The high sensitivity was attributed to the high electrocatalytic activity of Cu_2_O nanoparticles.

**Figure 11.**
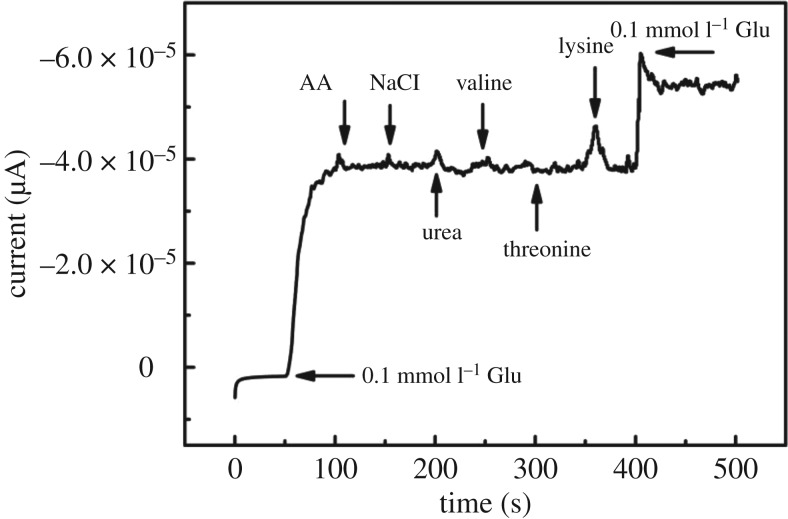
Amperometric responses of the Cu_2_O/HCFs-modified electrode to successive additions of glucose (0.1 mM), AA (0.01 mM) and NaCl (0.01 mM), urea (0.01 mM), valine (0.01 mM), threonine (0.01 mM), lysine (0.01 mM) in 0.1 M NaOH.

To evaluate the selectivity of the Cu_2_O/HCFs/GCE, seven possible interfering biomolecules, threonine, valine, lysine, glutamic acid, ascorbic acid (AA), urea and NaCl, which normally coexist with glucose in human blood were examined. The experimental results are shown in figure 11. Taking into consideration that the concentration of glucose is at least 30 times that of AA, NaCl, urea, valine, threonine and lysine, which is much higher than the concentrations of interfering species in human blood [36], thus, normal physiological levels of glucose (0.1 mM), AA (0.01 mM) and NaCl (0.01 mM), urea (0.01 mM), valine (0.01 mM), threonine (0.01 mM), lysine (0.01 mM), in 0.1 M NaOH solution by CV measurement. As can be seen from figure 11, our fabricating sensor demonstrates high selectivity and reliable anti-interference property by comparing the amperometric responses of other relevant electroactive species.

It is well known that the reproducibility and stability are also two important parameters for electrochemical sensors. The experimental results are shown in table 2. The reproducibility experiment of the Cu_2_O/HCFs/GCE was carried out in the 0.1 M NaOH solution by adding 0.1 mM glucose and measuring the current responses. In a series of five different electrodes prepared under the same condition, the relative standard deviation (RSD) is 5.64%, indicating that the Cu_2_O/HCFs/GCE can hence be a repeated preparation. The storage stability was evaluated at intervals by the measurement toward 0.1 mM glucose in the 0.1 M NaOH solution, and the electrode was stored at room temperature when not in use. The proposed Cu_2_O/HCFs/GCE retained about 89.73% of its initial response after 10 days, indicating the Cu_2_O/HCFs/GCE has a relatively stable electrochemical performance. Therefore, the Cu_2_O/HCF we prepared is an excellent candidate for the fabrication of stable, sensitive and specific sensors for the non-enzymatic detection of glucose.

**Table 2.** The current responses of reproducibility and repeated experiment.

performance testing	1	2	3	4	5
stability experiment	1.269 × 10^−4^	1.272 × 10^−4^	1.270 × 10^−4^	1.284 × 10^−4^	1.278 × 10^−4^
repeated experiment	1.416 × 10^−4^	1.339 × 10^−4^	1.327 × 10^−4^	1.295 × 10^−4^	1.269 × 10^−4^

## 4. Conclusion

In summary, we have presented the fabrication of Cu_2_O NPs-doped one-dimensional carbon hollow nanomaterial Cu_2_O/HCFs via thermal decomposition of a mixture of glucose and CuAc_2_ inside cylindrical nano-channels of the AAO template and further carbonization. The photos of SEM and TEM indicate that the diameter of the carbon hollow nanomaterials is about 60 nm, which is in accord with the size of the pores in the AAO templates. The Cu_2_O/HCFs can be explained. The XRD patterns that demonstrate Cu_2_O NPs have the face-centred cubic lattice. The Cu_2_O/HCFs/GCE show a wide linear range from 7.99 to 33.33 µM with a high sensitivity of 1218.3 µA cm^−2^ mM^−1^ and a detection limit down to 0.48 µM for glucose. The interfering species commonly presenting in the environment have no obvious effect on the oxidation of glucose on the Cu_2_O/HCFs. Owing to the ease of synthesis, good reproducibility and stability, Cu_2_O/HCFs become the promising microstructures for electrochemical biosensor devices of glucose.

## Supplementary Material

Figure;Supporting information

## Data accessibility

The datasets supporting this article have been uploaded as a part of the electronic supplementary material.
