# Supplementary material for: Template synthesis of the Cu2O nanoparticle-doped hollow carbon nanofibres and their application as non-enzymatic glucose biosensors
Source: R Soc Open Sci. 2018 Dec 12;5(12):181474. doi: 10.1098/rsos.181474 (PMC6304140; doi:10.1098/rsos.181474)
Supplement: Figure;Supporting information [file rsos181474supp1.doc]

**Supporting information**

Template synthesis of the Cu_2_O nanoparticles doped hollow carbon nanofibers and their application as nonenzymatic glucose biosensors

Yingjie Li, Renhao Cai, Renjiang Lü*, Lidi Gao,Shili Qin

*College of Chemistry and Chemical Engineering, Qiqihar University, Qiqihar, Heilongjiang, 161006, P. R. China,*

*Tel: +86 0452 2738251 ; Email:lvrenjiang123@163.com*


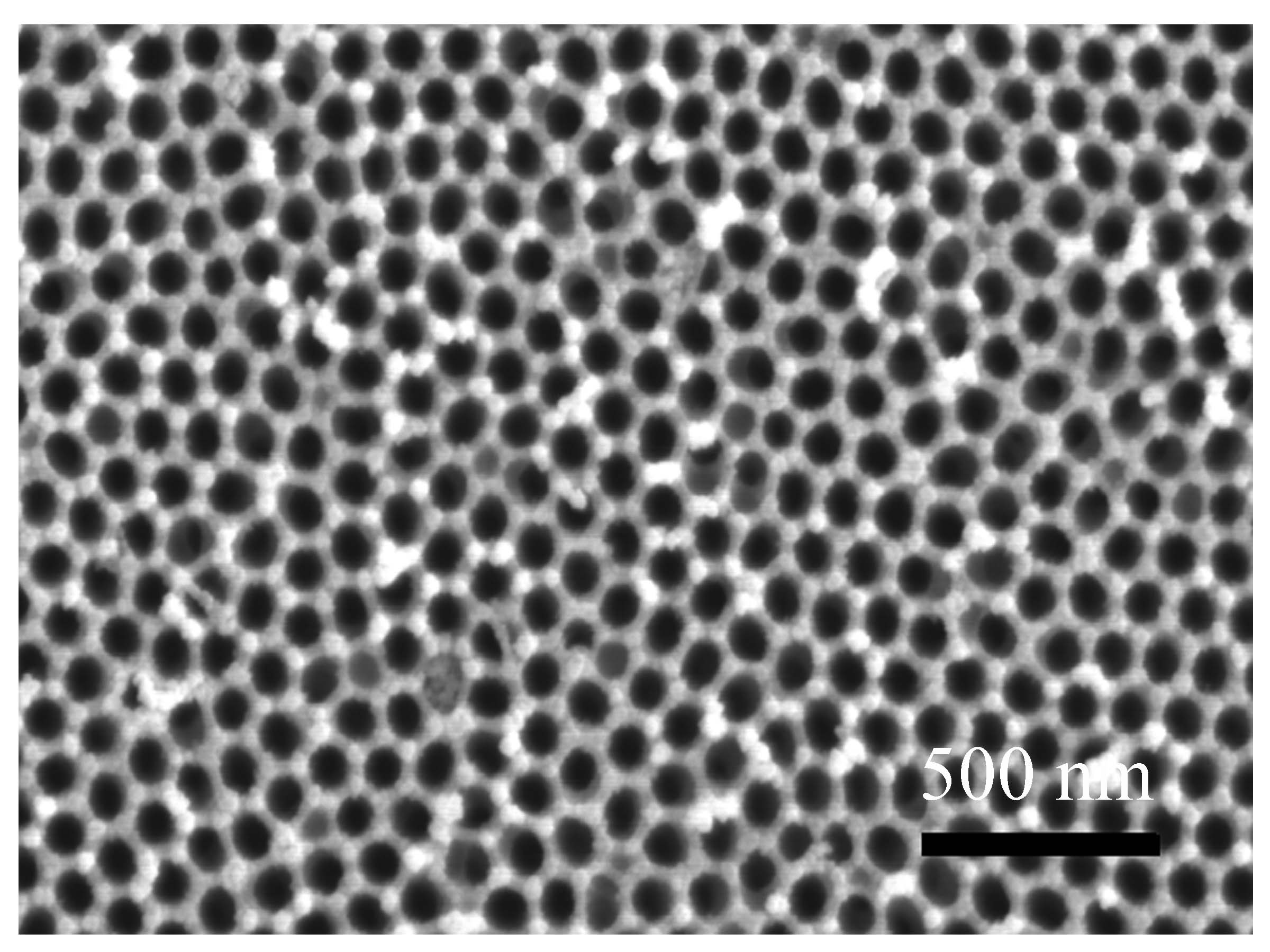


**Fig. S1** SEM images of the prepared AAO membrane with ~60 nm

pore diameters ( top view ).


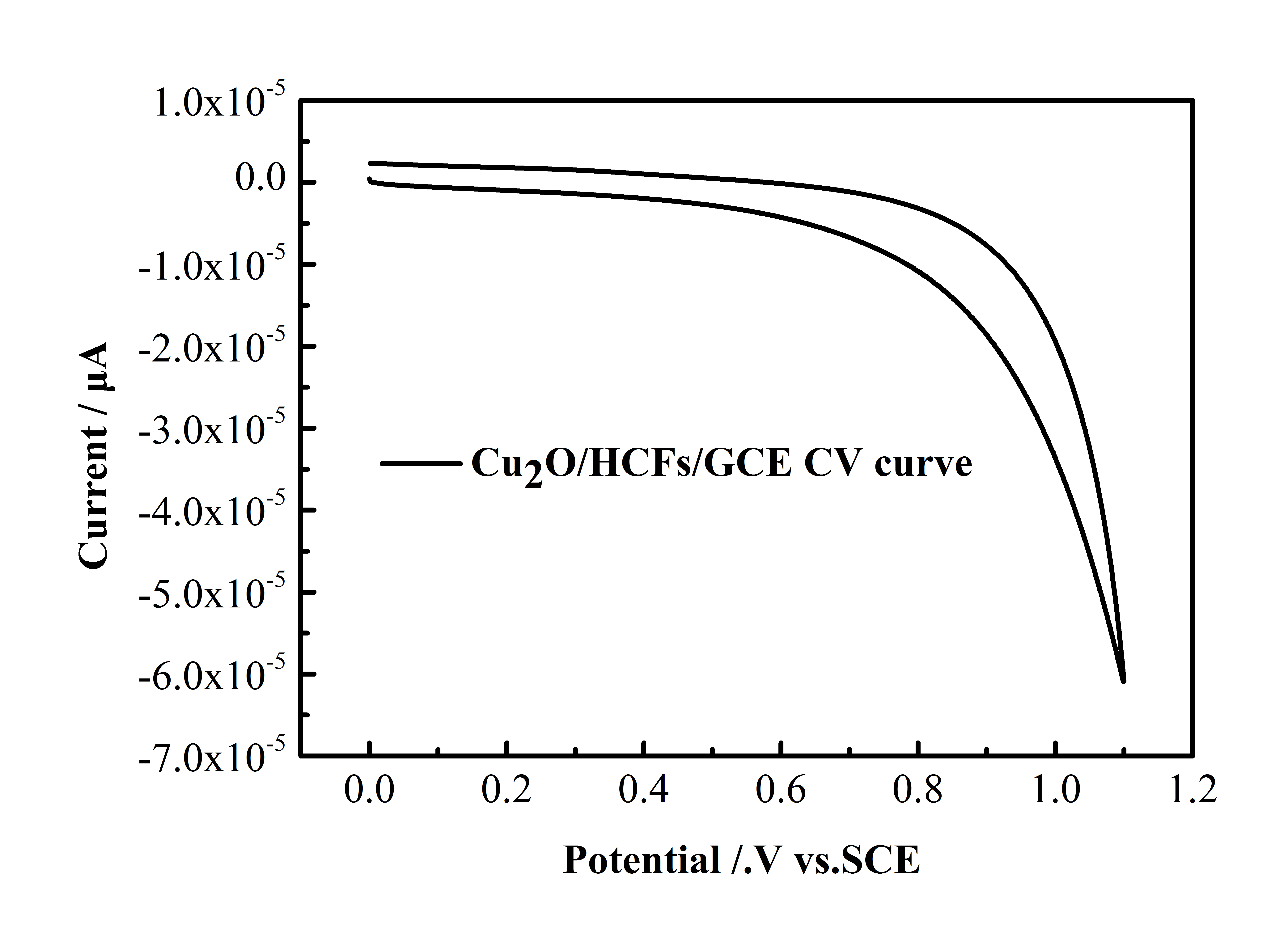


Fig.S2 CV curve of Cu_2_O/HCFs/GCE modified electrode for glucose detection


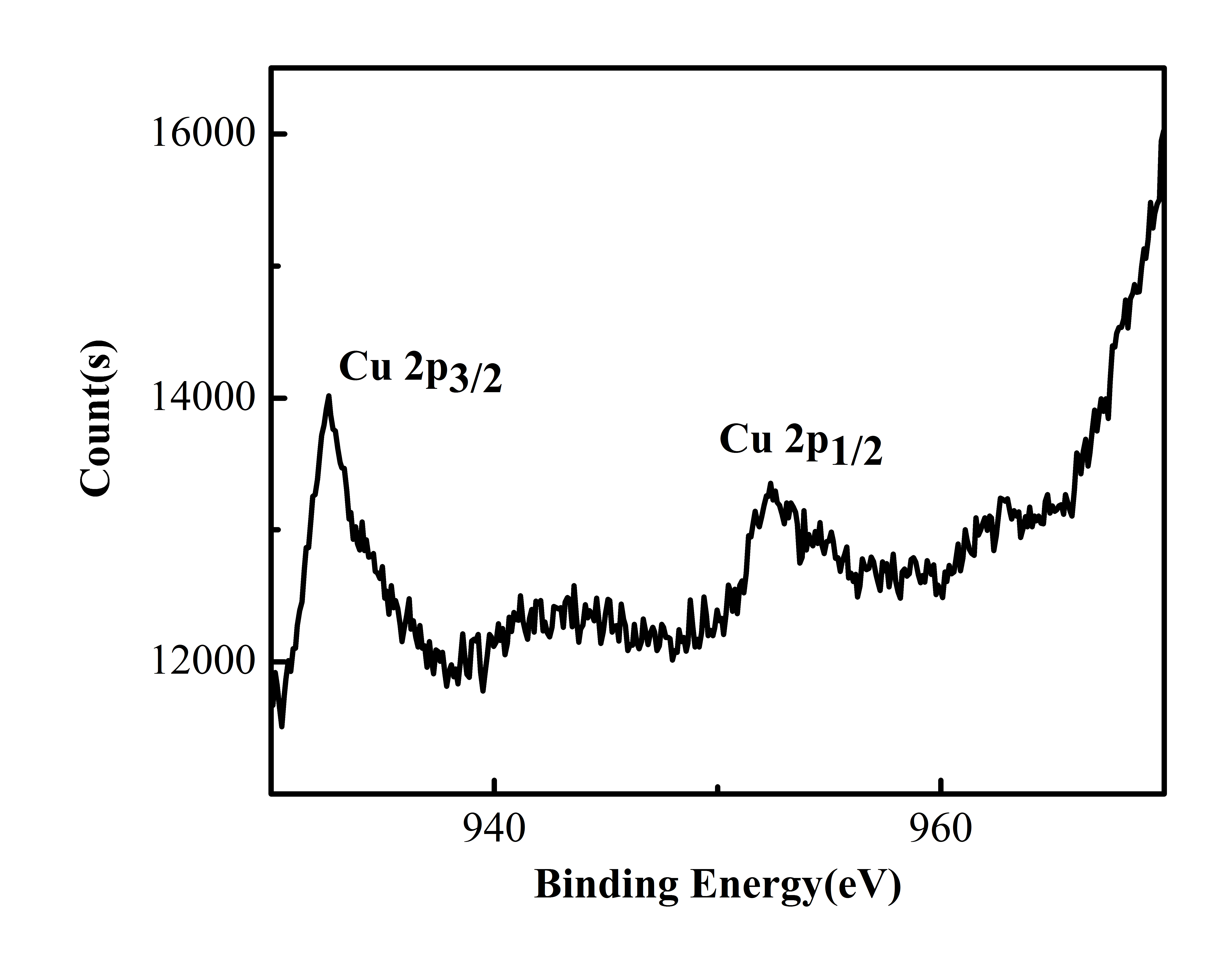

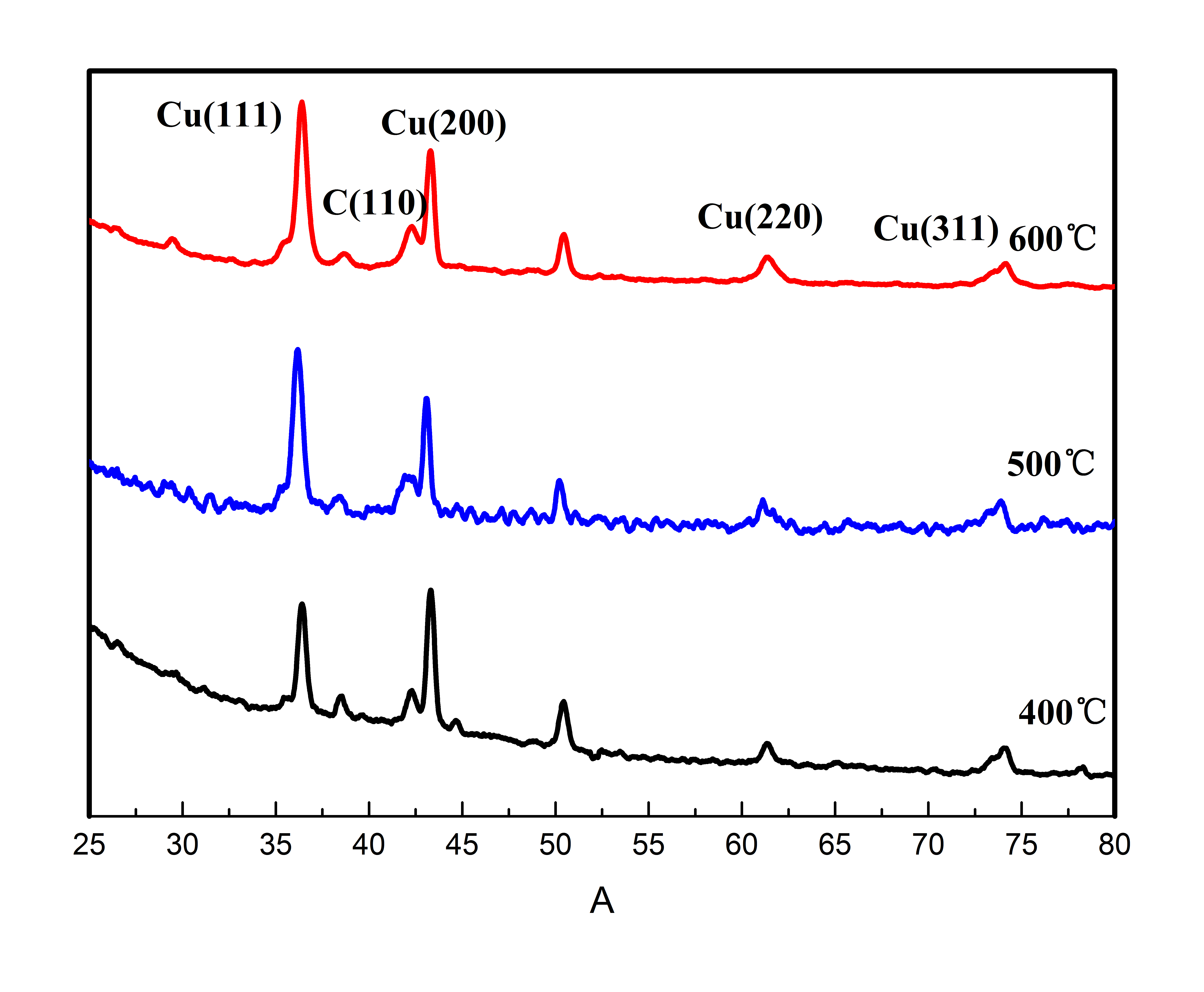


Fig.S3 The above two pictures prove that we successfully prepared Cu_2_O in the experiment.
